# An Emerging Role for OGDHL: From Mitochondrial Energy Metabolism to Neurodevelopmental Disorders

**DOI:** 10.3390/biology14121777

**Published:** 2025-12-12

**Authors:** Xian Liu, Guicheng Zhang, Decai Yu, Junhai Han

**Affiliations:** 1 School of Life Science and Technology, The Key Laboratory of Developmental Genes and Human Disease, Southeast University, 2 Dongda Road, Nanjing 210031, China; 2 State Key Laboratory of Pharmaceutical Biotechnology, Division of Hepatobiliary and Transplantation Surgery, Department of General Surgery, Affiliated Drum Tower Hospital Clinical College of Jiangsu University, Nanjing 210008, China; yudecai@nju.edu.cn; 3 Division of Hepatobiliary and Transplantation Surgery, Department of General Surgery, Nanjing Drum Tower Hospital, Affiliated Hospital of Medical School, Nanjing University, Nanjing 210008, China

**Keywords:** oxoglutarate dehydrogenase L, tricarboxylic acid cycle, mitochondrial metabolism, neurodevelopment disorders

## Abstract

**Simple Summary:**

The role of mitochondrial metabolism in neurodevelopment is increasingly recognized. Oxoglutarate dehydrogenase L (OGDHL), a brain-enriched rate-limiting enzyme in the tricarboxylic acid cycle, has been tightly linked to neurodevelopmental disorders, highlighting its essential role in neural processes. Although OGDHL has been extensively studied in liver and cancer biology, its functions in the brain are often overlooked. This review offers a comprehensive synthesis of current knowledge on OGDHL, including its discovery, molecular structure, and biological functions, and presents a detailed catalog of reported pathogenic mutations of OGDHL and their associated clinical phenotypes. By linking mitochondrial energy metabolism and neural pathogenesis, this review aims to advance our understanding of OGDHL’s functions in the nervous system and establish a foundation for elucidating the mechanisms underlying OGDHL-related neurological diseases.

**Abstract:**

The oxoglutarate dehydrogenase-like (OGDHL) gene encodes a brain-enriched, rate-limiting enzyme in the tricarboxylic acid cycle, playing an essential role in mitochondrial energy metabolism. Mutations in OGDHL are linked to a broad spectrum of neurodevelopmental disorders, characterized by developmental delay, intellectual disability, epilepsy, corpus callosum dysgenesis, and sensory deficits. This mini-review systematically summarizes the discovery, structural features, and molecular functions of OGDHL, and provides a comprehensive catalog of all reported pathogenic mutations and their clinical phenotypes. By linking mitochondrial energy metabolism and neural pathogenesis, this work positions OGDHL as a potential key regulator in neural development and function. Ultimately, this review aims to advance further research on OGDHL in the nervous system, enhance the understanding of metabolic regulation in neurodevelopment, and lay the groundwork for elucidating the mechanisms underlying OGDHL-related neurological diseases.

## 1. Introduction

Neuronal development and function require the continuous supply of adenosine triphosphate (ATP) to support neuronal differentiation, neurite growth, synaptic formation, and ion homeostasis [1]. Mitochondrial metabolism serves as the main energy source, providing ~93% of the total ATP used by neurons [2,3]. It is well documented that impairments in mitochondrial metabolism contribute to various neurodevelopmental, neurodegenerative, and psychiatric disorders, underscoring the importance of precisely controlled mitochondrial function across the central nervous system [4,5,6,7,8,9,10].

**Figure 1:**
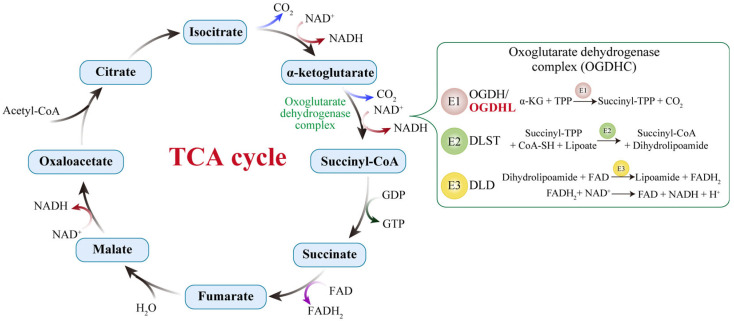
The TCA cycle and the catalytic reactions of the 2-oxoglutarate dehydrogenase complex (OGDHC). The OGDHC catalyzes the conversion of α-ketoglutarate into succinyl-CoA through the coordinated action of its three enzymatic components: E1 (OGDH), E2 (DLST), and E3 (DLD). In the brain, the isoenzyme OGDHL serves as an additional E1 component. The brain-specific OGDHL and the ubiquitously expressed OGDH exhibit differences in kinetics and calcium sensitivity, enabling neurons to precisely fine-tune carbon flux in response to synaptic activity or energy demands. When OGDHL activity is compromised, the oxidation of α-KG is impeded, leading to an accumulation of this TCA cycle intermediate, reduced generation of NADH/FADH_2_, insufficient substrate supply to the electron transport chain (ETC), and consequently, a decline in ATP synthesis.

The tricarboxylic acid (TCA) cycle is a central hub of mitochondrial metabolism, providing the main pathway that oxidizes carbohydrates, fats, and amino acids to obtain the energy to maintain cerebral functions [1]. The 2-oxoglutarate dehydrogenase complex (OGDHC) is the rate-limiting enzyme of the TCA cycle, influencing both ATP production and the synthesis of critical metabolic intermediates [11,12,13]. OGDHC, also known as the α-ketoglutarate dehydrogenase complex (KGDHC), comprises three catalytic enzyme components: E1o, thiamine diphosphate (ThDP)-dependent 2-oxo acid dehydrogenase; E2o, dihydrolipoyl acyl transferase; and E3, dihydrolipoyl dehydrogenase. These subunits work sequentially within the multienzyme complex to convert α-ketoglutarate (α-KG) into succinyl-CoA and generate ATP and NADH through oxidative phosphorylation [11,13,14] (Figure 1). Oxoglutarate dehydrogenase-like (OGDHL) is the isoenzyme of OGDH, which encodes the catalytic core subunit of the OGDHC. Both OGDH and OGDHL participate in the rate-limiting step that catalyzes the oxidative decarboxylation of 2-oxo acids. In contrast to the ubiquitously expressed OGDH, OGDHL exhibits a restricted expression profile, with particularly high levels in the central nervous system [15,16]. Mutations in OGDHL have been linked to a range of neurodevelopmental disorders, underscoring its specialized role in maintaining neuronal function and survival [17].

Despite growing interest in OGDHL, its specific contributions to neural development and disease pathogenesis remain incompletely characterized. This mini-review systematically summarizes the discovery and structural features of OGDHL, synthesizes its multifaceted roles within the nervous system and other tissues, and provides a detailed overview of the spectrum of OGDHL mutations identified to date. By integrating these insights, we aim to advance the understanding of OGDHL’s functions in the nervous system and establish a foundation for elucidating the mechanisms underlying OGDHL-related neurological diseases.

## 2. Discovery and Structural Characteristics of the OGDHL Protein

The human OGDHL gene, located on chromosome 10q11.23, comprises 22 exons and undergoes alternative splicing or 3′ processing to produce multiple transcript variants [18]. The initial clue of its existence came from the large-scale cDNA sequencing analyses of human and mouse genomes in the early 2000s [19,20,21]. Subsequent structure–function studies suggested that it encoded a functional enzyme [15]. This hypothesis received experimental validation in 2008 through work by Bunik and colleagues, who purified the native OGDHC from mitochondrial extracts of brain and heart tissue. Using nano-LC-MS/MS, they identified two non-overlapping peptide populations within the approximately 110 kDa band of brain OGDHC: one mapping to the canonical OGDH and another unique to OGDHL, each accounting for roughly 60% and 40% of the total signal, respectively. Further kinetic analyses revealed that brain OGDHC exhibited a biphasic substrate response, which correlated with the relative abundance of the two isoforms. These findings solidified OGDHL’s role as a bona fide catalytic component of the OGDHC [16].

**Figure 2:**
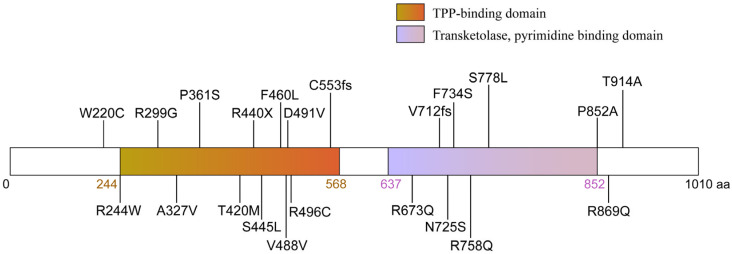
Representation of the domain structure and mutations of OGDHL. The OGDHL protein (1010 amino acids) contains a thiamine pyrophosphate (TPP)-binding domain and a pyrimidine-binding domain, both of which are catalytically essential. The diagram maps the 21 disease-associated mutations identified in patients, which include missense, frameshift, and nonsense mutations. These mutations predominantly cluster within or adjacent to key functional domains.

OGDHL encodes a 115-kDa protein that contains a thiamine pyrophosphate (TPP)-binding domain responsible for α-KG recognition, as well as a Transketolase, pyrimidine-binding domain that catalyzes the decarboxylation of α-KG to form succinyl-TPP (Figure 2). In silico structural modeling indicates that OGDHL forms a homodimer, with each subunit binding a TPP cofactor to create a substrate-binding pocket [17]. Comparative structural analyses reveal an 85% overall sequence similarity between OGDHL and OGDH, with most structural differences localized to the N- and C-terminal regions [15,16]. These regions are known to regulate the homo- and heterologous protein–protein interactions of 2-oxo acid dehydrogenases [22,23,24,25]. For instance, OGDHL may form tetramers bound to the E2o-formed core, whereas OGDH typically binds as dimers [16,26]. Additionally, some deletions in the N-terminal domain of OGDHL (~10 kDa) may contribute to the reduced affinity of brain OGDHC to E3 [16]. Hence, isoenzyme-specific protein–protein interactions may affect enzymatic kinetics or allosteric regulation that underlie the distinct functional properties and neural-specific roles of OGDHL.

## 3. Mutations in the Human OGDHL Gene and Associated Clinical Syndromes

In recent years, advances in high-throughput sequencing technologies, particularly whole-exome sequencing and whole-genome sequencing, have greatly facilitated the identification of pathogenic mutations in rare neurodevelopmental disorders, including those linked to OGDHL. To date, 21 distinct pathogenic variants in OGDHL have been reported across unrelated families [17,27,28], causing a spectrum of neurological and neurodevelopmental disorders, including epilepsy, hearing loss, visual impairment, gait ataxia, microcephaly, and hypoplasia of the corpus callosum. These can be classified into three groups according to the affected protein domain and mutation type: homozygous point mutations in the TPP-binding domain, homozygous point mutations in the transketolase domain, and compound heterozygous mutations. In this section, we provide a summary of the reported OGDHL mutations and their associated clinical phenotypes (Figure 2, Table 1).

Neurodevelopmental impairment represents the core phenotype in individuals with biallelic OGDHL mutations. Nearly all affected individuals exhibit varying degrees of developmental delay (DD) and/or intellectual disability (ID), emerging within months after birth. Severe cases, such as p.Arg244Trp, p.Arg440Ter, p.Ser445Leu and p.Phe460Leu variants, are characterized by global DD, ID, microcephaly, short stature, facial dysmorphism and motor delay; the milder forms, such as p.Pro852Ala, p.Thr914Ala variants, often achieve independent ambulation but exhibit learning difficulties [17,27]. Brain magnetic resonance imaging (MRI) reveals that OGDHL mutations lead to multifocal structural abnormalities. The most frequent observation, 11 of 29 documented cases, is corpus callosum hypoplasia, often accompanied by a constellation of posterior fossa anomalies, including ventriculomegaly, reduced cerebral white matter volume, and cerebellar and brainstem malformations [17,27].

Epilepsy represents another common neurological manifestation, occurring in approximately 9 of the 29 reported patients. Documented seizure types encompass infantile spasms, myoclonic-atonic seizures, and focal to bilateral tonic–clonic seizures. Sensory deficits are also frequently observed. Notably, about 28% (8/29) of patients exhibit bilateral profound or severe sensorineural hearing loss—a prevalence significantly higher than that reported in other metabolic neurodevelopmental disorders. Visual impairments are often severe and include optic atrophy, retinopathy, nystagmus, significantly reduced visual acuity, and roving eye movements [17,27]. Furthermore, a recent three-generation Chinese pedigree study has linked OGDHL to affective disorders. Carriers of the p.Asn725Ser variant presented with moderate depression, accompanied by structural abnormalities such as enlarged amygdala and cerebellar volumes, along with elevated blood glutamate levels [28]. This finding not only expands the phenotypic spectrum of OGDHL but also suggests its role in the mitochondrial–glutamate pathway and mood regulation. Importantly, it offers a monogenic disease model for the study of “metabolic depression.”

The impact of OGDHL mutations extends beyond the nervous system to involve skeletal, cardiovascular, and other systems. A notable proportion of patients (13/29) present with short stature and joint laxity. A patient with homozygous p.Arg758Gln mutation was reported to have hypertrophic cardiomyopathy and patent ductus arteriosus [27]. Additionally, individuals with p.Arg244Trp or p.Cys553Leufs16 variants displayed feeding difficulties and growth hormone deficiency [17]. A case of glioblastoma was also identified in a patient with homozygous p.Cys553Leufs16 [17]. These diverse clinical manifestations underscore the multifaceted biological functions of OGDHL.

We further compared the severity of clinical phenotypes caused by the mutations on different domains. Notably, mutations in the TPP-binding domain seem to lead to more severe clinical symptoms than those in the transketolase domain. Among these, p.244. Trp and p.Arg440Ter variants represent the most severe forms, with all carriers exhibiting global DD, ID, epilepsy or epileptic encephalopathy, spastic quadriplegia, and corpus callosum dysgenesis. Even heterozygous variants in this domain, such as the compound heterozygous p.Asp491Val and p.Arg496Cys, result in moderate motor delay and myoclonic discharges, indicating that single-allelic alterations can cause significant neurological dysfunction. In contrast, mutations in the transketolase domain generally lead to selective functional impairment rather than globally developmental phenotypes [17,27]. Within this group, 38% of patients display hearing impairment, only 25% develop epilepsy, and the prevalence of severe intellectual disability is markedly reduced. Occasional cases are associated with congenital heart disease or depression [17,28]. This domain-specific phenotypic divergence underscores the importance of delineating the precise role of each protein domain and developing mutation-specific disease models to elucidate the pathogenic mechanisms underlying OGDHL-related disorders.

## 4. The Functions of OGDHL in the Nervous System

Building on the genetic evidence that links OGDHL mutations to neurodevelopmental disorders, this section will discuss the multifaceted physiological and pathological functions of OGDHL in the nervous system.

### 4.1. Central Role in Energy Metabolism

The mammalian brain accounts for approximately 20% of the body’s total energy consumption despite representing only 2% of the total body mass [30]. Neurons rely almost exclusively on aerobic metabolism to generate ATP, supporting essential functions including synaptic formation and pruning, maintenance of resting membrane potentials, action potential propagation, and neurotransmitter recycling [1,31,32]. Within this metabolic framework, OGDHL serves as a critical regulatory component of the mitochondrial OGDHC, directly controlling TCA cycle flux. By catalyzing the irreversible oxidative decarboxylation of α-KG to succinyl-CoA, OGDHC drives the production of NADH, which fuels the electron transport chain at Complex I to establish the proton gradient essential for ATP synthesis [33,34,35]. The functional significance of OGDHL is further evidenced by its distinct expression pattern within the CNS. Consistent with the substantial energy demands of synaptic activity, OGDHL demonstrates enriched expression in regions of high synaptic density, including the cerebral cortex, caudate nucleus, and cerebellum. Furthermore, its expression exhibits neuronal specificity, being predominantly localized to neurons rather than glial cells [36]. Experimental evidence from neural-specific OGDHL mutation models confirms that reduced OGDHL expression compromises OGDHC activity, leading to significantly decreased oxygen consumption rates and impaired ATP production [17]. Consistent with these findings, OGDHL is downregulated in triple-transgenic Alzheimer’s mice, paralleling reduced OGDHC activity. Notably, OGDHL overexpression ameliorates cognitive deficits and Alzheimer’s-like pathology, potentially through Wnt/β-catenin signaling activation via upregulation of Wnt7B. This pathway modulation contributes to reduced neuroinflammation, amyloid plaque deposition, and tau hyperphosphorylation [37]. Furthermore, recent research has shed light on the involvement of OGDHL in Parkinson’s disease (PD) pathogenesis. OGDHL acts as one of the bottleneck enzymes in the TCA cycle in PD, which is downregulated in PD brains and models, leading to mitochondrial dysfunction and TCA cycle impairment. The resultant imbalance in the α-KG/fumarate ratio inhibits histone H3K4me3 demethylation. Elevated H3K4me3 binds to the promoter of the *SNCA* gene, promoting α-synuclein transcription and aggregation—a central pathological hallmark of PD. Importantly, the study suggests that correcting the abnormal metabolic flux in the TCA cycle via citrate supplementation may represent novel therapeutic strategies to mitigate α-synuclein pathology in PD [38]. These findings suggest that OGDHL is a key regulator of neuronal energy homeostasis, whose activity is crucial for modulating the ATP supply necessary to sustain the brain’s high-energy functions in both physiological and pathological states.

### 4.2. Biosynthesis of Neurotransmitters: Glutamate/GABA Cycle

**Figure 3:**
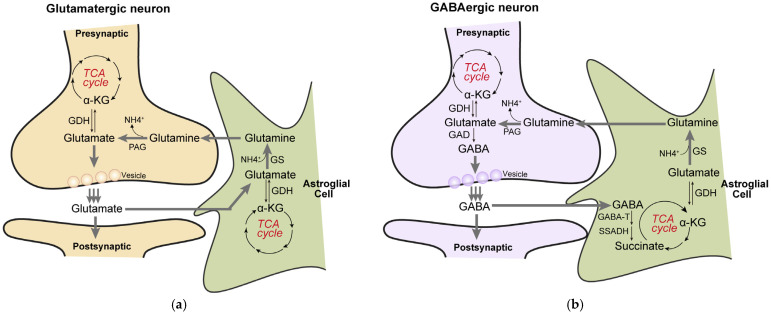
The TCA cycle is closely linked to the glutamate/GABA-glutamine cycle. (**a**), In glutamatergic neurons, α-ketoglutarate (α-KG) derived from the TCA cycle is converted to glutamate via glutamate dehydrogenase (GDH). The synthesized glutamate is released from presynaptic terminals into the synaptic cleft and is rapidly taken up by surrounding astrocytes through specific transporters. Within astrocytes, glutamate is converted to glutamine by glutamine synthetase (GS). The resulting glutamine is released into the extracellular space and transported back to neurons; meanwhile, a portion of glutamate can also be reconverted to α-KG through transamination or related reactions, thereby replenishing the TCA cycle. In neurons, glutamine is hydrolyzed back to glutamate by phosphate-activated glutaminase (PAG), completing a trans-cellular metabolic cycle. (**b**), In GABAergic neurons, glutamate is first decarboxylated by glutamate decarboxylase (GAD) to produce GABA. After release as an inhibitory neurotransmitter, GABA is similarly taken up by astrocytes. Within astrocytes, GABA undergoes a transamination reaction catalyzed by GABA transaminase (GABA-T), yielding succinic semialdehyde and glutamate. Succinic semialdehyde is then oxidized to succinate by succinic semialdehyde dehydrogenase (SSADH). Succinate enters the TCA cycle directly and, after a full turn of the cycle (including succinyl-CoA synthesis, substrate-level phosphorylation, and subsequent dehydrogenation/decarboxylation steps), is regenerated as α-KG. The newly formed α-KG can again be used for glutamate synthesis, thereby continuously replenishing the neuronal neurotransmitter precursor pool and maintaining homeostasis of energy-related metabolic intermediates.

Glutamate and γ-aminobutyric acid (GABA) are the main excitatory and inhibitory neurotransmitters, respectively, in the brain, and they are predominantly released from distinct neuronal pre-synaptic terminals. The glutamate/GABA-glutamine cycle is a major metabolic flux in the brain and the activity of the cycle is directly dependent on cerebral glucose metabolism and the TCA cycle [39,40,41,42]. The TCA cycle serves as a biosynthetic hub, with the cycle intermediates are diverted to primarily generate glutamate, GABA, and glutamine, thereby coordinating neurotransmitter synthesis, release, and recycling in a tightly coupled process between neurons and astrocytes [43,44,45,46,47]. Glutamate, as the most abundant excitatory neurotransmitter in neurons, is synthesized from α-KG by the glutamate dehydrogenase (GDH) [48]. By catalyzing the oxidative decarboxylation of α-KG to succinyl-CoA, OGDHC directs the carbon flux away from glutamate anabolism and toward catabolism, thereby regulating the availability of α-KG for neurotransmitter synthesis and influencing synaptic glutamate levels [35]. Following synaptic release, glutamate is taken up by surrounding astrocytes, converted to glutamine by glutamine synthetase, and shuttled back to neurons to be reconverted to glutamate, sustaining the cycle [46,49,50] (Figure 3a). Similarly, in GABAergic neurons, GABA is synthesized directly from glutamate. The metabolic fate of released GABA also converges on the TCA cycle, as it can be metabolized in astrocytes to succinate, which is then processed within the cycle to regenerate α-KG and glutamate, closing the metabolic loop [46] (Figure 3b). The fine-tuned metabolic coupling between glutamate and GABA synthesis not only sustains basal neurotransmission but is also crucial for dynamic synaptic interactions between excitatory and inhibitory neurons. For instance, a tight excitation-inhibition (E-I) balance involving the ubiquitous motif of reciprocally connected excitatory-inhibitory cells allows an alternation between fast excitation and delayed feedback inhibition to generate gamma oscillations [51,52]. The reliability of this oscillation is highly dependent on the timely supply of glutamate and GABA, which itself hinges on the metabolic capacity of the TCA cycle and the availability of α-KG. Given that OGDHL acts as a key regulator of α-KG flux and TCA cycle function, its activity may influence the stability and amplitude of gamma oscillations by modulating neurotransmitter precursor availability. Thus, through its role in sustaining the glutamate/GABA–glutamine cycle, OGDHL may contribute to the metabolic foundation of rhythmic network activity underlying sensory processing and cognitive functions, offering a potential mechanistic link between mitochondrial metabolism and higher brain functions.

Collectively, these findings propose OGDHL as a potential key regulator in maintaining the excitatory-inhibitory balance via its role in the TCA cycle. Although direct mechanistic evidence remains limited, clinical observations support this link. For instance, the family members carrying OGDHL mutations exhibit elevated glutamate levels in the brain compared to healthy members. Affected individuals presented with depressive symptoms and associated brain imaging alterations, including an enlarged left amygdala and a slight volume increase in the left cerebellum, suggesting that OGDHL deficiency may lead to chronic glutamate imbalance, which could contribute to the underlying pathophysiology [28].

## 5. The Roles of OGDHL in Other Systems

Besides the nervous system, OGDHL also plays an important role in other systems. The function of OGDHL has been extensively studied in the liver, particularly in the context of hepatocellular carcinoma (HCC). OGDHL expression is downregulated in HCC due to promoter hypermethylation and copy number loss, which correlates with advanced tumor stage, poor prognosis, and more frequent tumor recurrence [53]. Therefore, OGDHL has been regarded as a prognostic biomarker for liver cancer patients [54]. Functionally, OGDHL silencing drives metabolic reprogramming by suppressing the activity of the OGDHC. This inhibition shifts glutamine-derived α-KG away from oxidative metabolism in the TCA cycle towards reductive carboxylation. This metabolic rewiring promotes de novo lipogenesis and supports the cellular antioxidant system, thereby sustaining tumor growth and survival. Furthermore, OGDHL depletion activates mTORC1 signaling in an α-KG-dependent manner, further amplifying lipogenesis. Thus, the downregulated OGDHL, acting both as an OGDHC suppressor and an mTORC1 activator, fine-tunes this metabolic rewiring to robustly promote the lipogenic program [53]. Beyond its canonical metabolic role, OGDHL exhibits a non-canonical function by localizing to the nucleus and inducing DNA damage independently of its enzymatic activity [55]. Mechanistically, nuclear OGDHL binds to CDK4 and inhibits its phosphorylation by CDK-activating kinase, leading to the downregulation of E2F1 signaling, a master regulator of cell cycle progression and nucleotide synthesis. The suppression of E2F1, in turn, reduces the synthesis of nucleotides, causing deoxyribonucleotide triphosphate (dNTP) depletion and ultimately triggering DNA damage, thereby inhibiting HCC progression [55]. This discovery significantly expands the functional repertoire of OGDHL beyond metabolism, siting it as a direct regulator of genomic integrity in cancer cells. The multifaceted roles of OGDHL in the liver-orchestrating mitochondrial metabolism, cellular signaling, and nuclear genome stability-provide a compelling paradigm for understanding how metabolic enzymes can possess diverse functions across different cellular compartments. Importantly, these current findings in liver highlight OGDHL not only as a critical prognostic marker but also a promising therapeutic target. Promising strategies emerging include AAV-mediated OGDHL gene therapy, which has been shown in animal models to effectively suppress tumor growth and prolong survival. Additionally, targeting the resulting glutamine metabolic vulnerability with glutaminase inhibitors have been demonstrated synergistic potential with standard therapies like sorafenib [53]. Based on its non-canonical function, CDK4/6 or PARP inhibitors could also be strategically deployed in OGDHL-low tumors to inhibit HCC progression [55]. Together, these insights offer novel directions for precise intervention in HCC.

OGDHL is also essential for cardiac development and contractility. Integrated proteomic and transcriptomic analyses have identified OGDHL as a hub gene that exhibits markedly upregulated expression during heart maturation [56]. Deletion of OGDHL in primary cardiomyocytes impairs mitochondrial respiration, glycolysis, and overall metabolic efficiency, driving the cells towards a quiescent state [56]. This link between OGDHL and cardiac energy metabolism is further supported by the observation that deficiency in the zinc-finger protein ZBTB20 attenuates the developmental activation of mitochondrial genes, including OGDHL, leading to reduced ATP production, compromised mitochondrial complex I activity, and ultimately, cardiac contractile dysfunction [57]. Moreover, OGDHL has been shown to regulate succinate metabolism and served as an important ROS generator in cardiac mitochondria. Under pathological stress conditions such as myocardial ischemia–reperfusion (I/R) injury, both the expression and activity of OGDHL are significantly elevated, contributing to mitochondrial ROS production and exacerbating injury. Notably, magnetic vagus nerve stimulation (mVNS) has been demonstrated to alleviate myocardial I/R injury by suppressing OGDHL expression through the M_2_AChR/OGDHL/ROS axis, ultimately inhibiting pyroptosis and relieving mitochondrial damage [58,59]. Additionally, in models of heart failure with preserved ejection fraction (HFpEF), abnormal abundances of OGDHL mRNA and protein have been observed in cardiac biopsies, while osteopontin deletion has been found to restore OGDHL expression, improve mitochondrial function, and normalize diastolic parameters [60]. OGDHL is also associated with myocardial fibrosis of dilated cardiomyopathy (DCM) and probably serves as a biomarker for myocardial remodeling in patients with DCM [61]. These findings indicate that OGDHL may represent a promising therapeutic target for multiple cardiovascular diseases. Its activity could be therapeutically enhanced in settings of impaired energy metabolism, while carefully moderated under conditions of exacerbated oxidative stress. Future research could explore specific OGDHL modulators or multimodal interventions combined with neuromodulation (such as vagus nerve stimulation), offering novel therapeutic strategies for cardiovascular diseases.

In the renal system, OGDHL is involved in maintaining metabolic homeostasis and preventing fibrosis. Studies indicate that the transcriptional repressor REST is upregulated in chronic kidney disease, where it directly binds to the OGDHL promoter and represses its transcription. This repression disrupts mitochondrial energy metabolism and fatty acid oxidation in renal tubular epithelial cells, thereby promoting the progression of renal fibrosis [62]. These findings suggest that restoring OGDHL function may represent a promising therapeutic strategy for mitigating pathology associated with chronic kidney disease.

OGDHL is also associated with the pathogenesis and progression of various cancers. Beyond hepatocellular carcinoma (HCC), studies have demonstrated that OGDHL expression is frequently silenced by cancer-specific promoter methylation in lung, breast, cervix, esophagus, pancreas, renal cell carcinoma and colon cancers [53,63,64,65,66,67,68]. Mechanistically, Sen et al. reported that OGDHL inactivation promotes cervical tumor proliferation by activating the AKT signaling pathway. Conversely, forced expression of OGDHL in cervical cancer downregulates AKT signaling, reduces caspase-3-mediated phosphorylation of NF-κB, enhances ROS production, which in turn induces apoptosis and inhibits cancer cell proliferation and metastasis [66]. In clear cell renal cell carcinoma (ccRCC), Shi et al. revealed that low OGDHL expression negatively regulates FASN transcription through TFAP2A, leading to ERK pathway activation and enhanced lipid synthesis [67]. Additionally, Liu et al. showed that an OGDHL-mediated miR-214/TWIST1 negative feedback loop suppresses the growth and metastasis of pancreatic cancer [68]. These findings collectively highlight the tumor-suppressive role of OGDHL across diverse cancer types.

## 6. Future Perspectives

While current findings indicate OGDHL as a critical player in mitochondrial metabolism and neurodevelopmental pathogenesis, significant gaps remain in our mechanistic understanding. Looking ahead, several critical questions and research directions warrant further investigation.

Firstly, current research on OGDHL has been largely confined to invertebrate models such as Drosophila and Zebrafish, which limits our pathophysiological understanding. There is an urgent need to develop more physiologically relevant models, including patient-derived iPSCs (particularly cerebral organoids) and knock-in mice carrying patient-specific OGDHL mutations. These tools will not only provide deeper mechanistic insights but also serve as indispensable platforms for drug screening and therapeutic development.

Second, the cellular mechanisms by which OGDHL influences neuronal development and function remain largely unknown. Given its established role in inhibiting proliferation in various cancers, a compelling question arises: does OGDHL similarly regulate the proliferation and differentiation of neural progenitor cells during neurogenesis? Dysregulation of this process could underlie the microcephaly observed in severe OGDHL deficiency. Furthermore, although OGDHL is indicated to function in the glutamate/GABA cycle, its direct impact on synaptic plasticity and the excitation-inhibition (E-I) balance, and whether this role exhibits regional and neuronal specificity remains unclear. While OGDHL is known to be enriched in the nervous system, its precise expression across brain regions, cell types, and developmental stages remains poorly mapped. Establishing spatiotemporal expression atlas across brain regions and neuronal subtypes, combined with conditional knockout models in mammals, will help clarify how OGDHL loss disrupts circuit assembly and leads to symptoms such as epilepsy and intellectual disability. Future studies should therefore prioritize key developmental windows, cell-type–specific roles, and synaptic consequences of OGDHL deficiency, which together will elucidate how OGDHL regulates neurodevelopmental processes—from neurogenesis to synaptic maturation—and how its dysfunction leads to disease.

Third, our understanding of OGDHL’s molecular function remains incomplete. Beyond its canonical metabolic function in cytoplasm, emerging evidence from cancer studies indicates that OGDHL can translocate to the nucleus, where it binds to CDK4 and suppresses its phosphorylation, leading to downregulation of the E2F1 signaling pathway and ultimately inducing DNA damage [55]. Notably, the DNA damage response is also critical for neurodevelopment, and its dysregulation may contribute to neurodegeneration or developmental disorders [69,70]. The ability of OGDHL to inhibit CDK4–E2F1 signaling and deplete dNTP pools, as demonstrated in HCC, may similarly affect neural progenitor proliferation and survival [71,72]. Future studies should investigate whether OGDHL’s nuclear function is conserved in neural cells and whether disease-associated mutations disrupt its nuclear localization signal, thereby altering its subcellular distribution. If validated, these parallels would suggest that therapeutic strategies aimed at restoring or activating OGDHL—such as AAV-mediated gene delivery or small-molecule enhancers—could hold promise not only in oncology but also in neurological disorders characterized by OGDHL deficiency. On the other hand, the fact that domain-specific mutations cause divergent clinical phenotypes strongly implies that these structural modules support distinct cellular functions. Systematically profiling the interactomes of OGDHL across different cellular compartments and within its specific domains would be beneficial for uncovering novel molecular functions of OGDHL. Such characterization is essential to elucidate the pathogenic mechanisms underlying patient mutations and to explain how mutations in different domains result in distinct clinical severities.

Fourth, it is unknown why the brain requires two paralogous enzymes, OGDH and OGDHL, for the same catalytic step. The inability of OGDHL to compensate for the loss of OGDH, and vice versa, suggests their distinct molecular and physiological functions. We speculate that there might exist a refined regulatory strategy in the energy-demanding brain that extends beyond basic α-KG decarboxylation. Early kinetic analyses of brain OGDHC provide important clues [16]. OGDH exhibits high substrate affinity (Km ≈ 0.07 mM), securing basal TCA cycle flux under low α-KG conditions, whereas OGDHL has a markedly lower affinity (Km ≈ 0.40 mM) and is substantially engaged only when α-KG levels rise, such as during neuronal excitation. This “two-speed” design allows the complex to operate efficiently across a wide range of substrate concentrations, supporting both routine metabolism and peak energetic demands. Structurally, both isoforms are stably co-assembled within the same OGDHC in an approximate 60:40 ratio (OGDH:OGDHL), enabling rapid, substrate-sensitive switching without the need for complex reassembly. Furthermore, evidence suggests that OGDHL may interact more weakly with the E3 component [16], which could attenuate ROS-generating side reactions during high metabolic activity. Thus, this bipartite architecture likely helps neurons balance energy production with redox protection, thereby stabilizing bioenergetic and excitotoxic homeostasis. Therefore, to clarify the mechanistic basis of their functional specialization, future studies should focus on characterizing their precise kinetic properties, subcellular localization, and neuronal interaction networks, ultimately elucidating how OGDH and OGDHL interact at the cellular level to fine-tune mitochondrial function in response to metabolic state.

Lastly but importantly, rare diseases present enduring challenges in medicine, characterized by small patient cohorts and highly variable clinical manifestations. Conventional drug development is often hampered by insufficient clinical data, leading to extended timelines and elevated costs. Advances in genetics, however, are now offering promising new avenues for treatment of rare diseases. To date, 21 distinct pathogenic variants in OGDHL have been associated with a spectrum of neurological and neurodevelopmental disorders. These findings not only benefit for elucidating the pathogenic mechanisms underlying specific mutations but also establish a foundation for personalized medicine. Emerging genomic tools, particularly CRISPR/Cas9-based base editing, hold great potential for precisely correcting disease-causing mutations, and in vivo base editing has been successfully applied in several disease models [73,74,75]. Notably, recent work by Zilong Qiu and Lin Cheng’s team have showed that in vivo base editing could accurately correct autism-associated mutations in the brain and alleviate core symptoms in mouse models [76]. This progress significantly enriches the genetic engineering toolkit and represents a major advance toward genomics-based personalized medicine for neurodevelopmental disorders, including those related to OGDHL.

## 7. Conclusions

The role of mitochondrial energy metabolism in neurodevelopment has garnered increasing attention in recent years. Accumulating evidence demonstrates that mitochondrial maturation and metabolic activity are essential for neuronal differentiation, maturation, and functional maintenance [77,78,79]. Mitochondrial dysfunction and brain energy metabolism impairment have been identified in various neurological disorders, including Leigh syndrome, autism spectrum disorder, epilepsy, and dystonia [80,81,82], yet the underlying mechanisms linking mitochondrial dysfunction to neurodevelopmental impairment remain largely elusive. OGDHL, as a brain-enriched, key rate-limiting enzyme in the TCA cycle, governs energy production, neurotransmitter homeostasis, and cell proliferation. OGDHL mutations have been associated with the expanding spectrum of clinical phenotypes, ranging from severe neurodevelopmental disorders to affective symptoms and multi-systemic involvement, underscoring their multifaceted functions in the nervous system. This review has systematically summarized current knowledge of OGDHL, including its discovery, molecular structure, and biological functions, while providing a detailed catalog of reported pathogenic mutations and their associated clinical phenotypes. By integrating OGDHL’s roles in mitochondrial energy metabolism and neural development, this work aims to advance the research of OGDHL in nervous system and deepen our understanding of the metabolic regulation of neurodevelopment.

## Figures and Tables

**Table 1:** A catalog of the reported OGDHL mutations.

Domain	Mutation	Amino Acid	Family	Clinical Symptoms
TPP-binding domain	c.730 A>T	Missense mutation, p.R244W	Family5	global DD and ID, spastic, optic atrophy, quadriplegic, microcephaly, corpus callosum hypoplasia
TPP-binding domain	c.895 A>T	Missense mutation, p.R299G	Family7	moderate to severe ID, mild nystagmus
TPP-binding domain	c.980 C>T	Missense mutation, p.A327V	Family11	motor delay and borderline ID, hyporeflexia, FTT
TPP-binding domain	c.1081 C>T	Missense mutation, p.P361S	Family6	Moderate ID, focal tonic seizures, hypertonia, ataxia, NDD, corpus callosum hypoplasia
TPP-binding domain	c.1259 C>T	Missense mutation, p.T420M	Family1,2,3	Microcephaly, short stature, FTT, DD and ID, Hypotonia, Sensorineural hearing loss (family1), NDD
TPP-binding domain	c.1318 C>T	Non-sense mutation, p.R440X	Family8	global DD and ID, hypotonia, GA, hearing and vision deficit, developmental and epileptic encephalopathy
TPP-binding domain	c.1334 C>T	Missense mutation, p.S445L	Family5	Severe DD, severe to profound ID, Short stature, FTT, seizures, NDD, corpus callosum hypoplasia
TPP-binding domain	c.1380 C>G	Missense mutation, p.F460L	Family10	Severe DD, Generalized tonic–clonic seizures, spastic quadriplegia, Thin corpus callosum, NDD
TPP-binding domain	c.1658 delG	Frameshift mutation from C553, p. C553L fs * 16	Family6	mild ID, glioma, scaphocephaly
transketolase domain	c.2133 delA	Frameshift mutation from V712, p. V712S fs * 77	Family9	severe to profound Non- syndromic hearing loss
transketolase domain	c.2174 A>G	Missense mutation, p.725N>S	Family1	Depressive Disorder
transketolase domain	c.2273 G>A	Missense mutation, p.758R>Q	Family12	Congenital heart defects
transketolase domain	c.2333 C>T	Missense mutation, p.778S>L	Family9	severe DD and ID, microcephaly, corpus callosum hypoplasia
transketolase domain	c.2554 C>G	Missense mutation, p.852P>A	Family1	mild DD, mild GA, hearing deficit
C-terminal	c.2606 G>A	Missense mutation, p.869R>Q	Family4	short stature, FTT, Hypotonia, Moderate ID, NDD
C-terminal	c.2740 A>G	Missense mutation, p.914T>A	Family7	short stature, FTT, mild DD, Right eye esotropia, suspected hearing difficulty
N-terminal TPP-binding domain	c.660 G>C/ c.1472 A>T	Compound heterozygous single-nucleotide variants, p.220W>C/p.491D>V	Family4	global DD, tonic–clonic, nystagmus, hypotonia, developmental and epileptic encephalopathy
N-terminal TPP-binding domain	c.660 G>C/ c.1486 C>T	Compound heterozygous single-nucleotide variants, p.220W>C/p.496R>C	Family8	Mild motor delay, ADHD, Myoclonic-astatic epilepsy, hypotonia, DD
TPP-binding domain transketolase domain	c.980 C>T/ c.2201 T>C	Compound heterozygous single-nucleotide variants, p.327A>V/p.734F>S	Family3	myoclonic-atonic, tonic–clonic, status epilepticus, developmental and epileptic encephalopathy
TPP-binding domain transketolase domain	c.1464 T>C/ c.2018 G>A	Compound heterozygous single-nucleotide variants, p.488V>V/p.673R>Q	Family2	mild DD, GA, spasticity, retinopathy, dysmorphic features

* All mutations listed were identified in the cited references [17,27,28,29].

## Data Availability

No new data were created or analyzed in this study. Data sharing is not applicable to this article.

## Abbreviations

The following abbreviations are used in this manuscript:

α-KG	Alpha-ketoglutarate
AD	Alzheimer’s Disease
ADHD	Attention-Deficit/Hyperactivity Disorder
AKT	Protein Kinase B
ALS	Amyotrophic Lateral Sclerosis
ASD	Autism Spectrum Disorder
ATP	Adenosine Triphosphate
ccRCC	Clear Cell Renal Cell Carcinoma
CDK4	Cyclin-Dependent Kinase 4
CNS	Central Nervous System
dNTP	Deoxyribonucleotide Triphosphate
DCM	Dilated Cardiomyopathy
DD	Developmental Delay
DLST	Dihydrolipoamide Succinyltransferase
DLD	Dihydrolipoamide Dehydrogenase
E1o	2-oxoglutarate dehydrogenase
E2o	Dihydrolipoamide Succinyltransferase
E3	Dihydrolipoamide Dehydrogenase
ERK	Extracellular Signal-Regulated Kinase
FASN	Fatty Acid Synthase
FTT	Failure to Thrive
GA	Gait Ataxia
GABA	Gamma-Aminobutyric Acid
GDH	Glutamate Dehydrogenase
HFpEF	Heart Failure with Preserved Ejection Fraction
HCC	Hepatocellular Carcinoma
ID	Intellectual Disability
I/R	Ischemia–Reperfusion
miR-214	MicroRNA-214
MRI	Magnetic Resonance Imaging
mTORC1	Mechanistic Target of Rapamycin Complex 1
mVNS	Magnetic Vagus Nerve Stimulation
NADH	Nicotinamide Adenine Dinucleotide (reduced form)
NDD	Neurodevelopmental Disorder
NF-κB	Nuclear Factor Kappa B
OGDHC	2-Oxoglutarate Dehydrogenase Complex
OGDH	2-Oxoglutarate Dehydrogenase
OGDHL	Oxoglutarate Dehydrogenase-Like
PD	Parkinson’s disease
REST	RE1-Silencing Transcription Factor
ROS	Reactive Oxygen Species
TCA	Tricarboxylic Acid Cycle
TFAP2A	Transcription Factor AP-2 Alpha
ThDP	Thiamine Diphosphate
TPP	Thiamine Pyrophosphate
TWIST1	Twist Family BHLH Transcription Factor 1
Wnt7B	Wingless-type MMTV Integration Site Family, Member 7B
ZBTB20	Zinc Finger and BTB Domain Containing 20
